# Precise mapping and dynamics of tRNA-derived fragments (tRFs) in the development of *Triops cancriformis* (tadpole shrimp)

**DOI:** 10.1186/s12863-015-0245-5

**Published:** 2015-07-14

**Authors:** Yuka Hirose, Kahori T. Ikeda, Emiko Noro, Kiriko Hiraoka, Masaru Tomita, Akio Kanai

**Affiliations:** Institute for Advanced Biosciences, Keio University, Tsuruoka, 997-0017 Japan; Systems Biology Program, Graduate School of Media and Governance, Keio University, Fujisawa, 252-8520 Japan; Faculty of Environment and Information Studies, Keio University, Fujisawa, 252-0882 Japan

**Keywords:** Transfer RNA, tRNA-derived fragment, Deep sequencing analysis, Development, Tadpole shrimp

## Abstract

**Background:**

In a deep sequencing analysis of small RNAs prepared from a living fossil, the tadpole shrimp *Triops cancriformis*, a 32-nt small RNA was specifically detected in the adult stage. A nucleotide sequence comparison between the 32-nt small RNA and predicted tRNA sequences in the draft nuclear genomic DNA showed that the small RNA was derived from tRNA^Gly^(GCC). To determine the overall features of the tRNA-derived fragments (tRFs) of *T. cancriformis*, the small RNA sequences in each of the six developmental stages (egg, 1st − 4th instar larvae, and adult) were compared with the mitochondrial and nuclear tRNA sequences.

**Results:**

We found that the tRFs were derived from mitochondrial and nuclear tRNAs corresponding to 16 and 39 anticodons, respectively. The total read number of nuclear tRFs was approximately 400 times larger than the number of mitochondrial tRFs. Interestingly, the main regions in each parental tRNA from which these tRFs were derived differed, depending on the parental anticodon. Mitochondrial tRF^Ser^(GCU)s were abundantly produced from the 5’ half regions of the parental tRNA, whereas mitochondrial tRF^Val^(UAC)s were mainly produced from the 3’ end regions. Highly abundant nuclear tRFs, tRF^Gly^(GCC)s, tRF^Gly^(CCC)s, tRF^Glu^(CUC)s, and tRF^Lys^(CUU)s were derived from the 5’ half regions of the parental tRNAs. Further analysis of the tRF read counts in the individual developmental stages suggested that the expression of mitochondrial and nuclear tRFs differed during the six stages. Based on these data, we precisely summarized the positions of the tRFs in their parental tRNAs and their expression changes during development.

**Conclusions:**

Our results reveal the entire dynamics of the tRFs from both the nuclear and mitochondrial genomes of *T. cancriformis* and indicate that the majority of tRFs in the cell are derived from nuclear tRNAs. This study provides the first examples of developmentally expressed mitochondrial tRFs.

**Electronic supplementary material:**

The online version of this article (doi:10.1186/s12863-015-0245-5) contains supplementary material, which is available to authorized users.

## Background

It is well known that transfer RNAs (tRNAs) are noncoding short RNAs of 70–100 nucleotides (nt) and are involved in the translation process as adapter molecules between the amino acids and the corresponding codons in the template mRNAs. In the last 10 years, several groups, including our own, have reported that particular tRNA genes, especially in the Archaea and primitive Eukaryota, are disrupted in unique ways: multiple-intron-containing tRNAs [1, 2], split tRNAs [3–5], tri-split tRNAs [4], and permuted tRNAs [5–8]. It is also accepted that even the tRNA molecules themselves are fragmented posttranscriptionally in many species, and these fragmented small RNAs are known as tRFs [9–18]. At the outset of tRF research, the greatest concern was that these fragments might simply be the degradation products of mature tRNAs. However, at least some tRFs appear to be biologically functional, based on the following observations: (a) tRFs are not always derived from abundant cellular tRNAs, and the numbers of tRFs do not correlate with the gene copy numbers of the parental tRNAs; (b) their fragmentation patterns are dependent on the parental tRNA anticodons; (c) the fragmentation patterns can change according to developmental stage or cellular conditions; and (d) some tRFs are bound to Argonaute/Piwi proteins, well-known components of the RNA-induced silencing complex [11, 17, 19]. In terms of the functions of tRFs, it has been reported that the conditional depletion of tRNAs by their conversion to tRFs might be related to the downregulation of protein synthesis [20]. Moreover, angiogenin-induced tRFs themselves inhibit the initiation of translation [10]. Other studies have suggested that at least some tRFs are involved in the regulation of gene silencing, in the same way as miRNAs, because tRFs bind to Argonaute/Piwi proteins [12, 15] and the generation of the tRFs is reported to be Dicer-dependent [21, 22]. Therefore, the tRFs have their own specific functions (including in RNA silencing), other than as parts of adapter molecules in translation.

Recently, we investigated the microRNAs (miRNAs) of the nonmodel species *Triops cancriformis* (tadpole shrimp) [23]. This organism is called a “living fossil” because its morphological form has not changed in almost 200 million years. miRNAs are members of the noncoding small RNAs are approximately 22 nt and regulate the expression of target messenger RNAs (mRNAs), mainly at the posttranscriptional level [24]. We used deep sequencing to analyze small RNA libraries from the six different developmental stages of *T. cancriformis* (egg, 1st–4th instars, and adult), and also analyzed the organism’s nuclear genomic DNA with deep sequencing. The aim of the present study was to survey the entire dynamics of tRFs using these genomic data and a series of transcriptomic data. We initially determined the set of tRNA genes encoded in both the mitochondrial and nuclear genomes of *T. cancriformis*. Using deep sequencing data from the small RNA fraction of the organism, we then precisely mapped the tRFs onto these genomes. Our results showed that the tRFs were derived from the mitochondrial and nuclear tRNAs corresponding to 16 and 39 specific anticodons, respectively. This study provides the first examples of developmentally expressed mitochondrial tRFs. Interestingly, the main regions in the parental tRNAs from which the mitochondrial tRFs are derived differ greatly, depending on the parental anticodon. However, most of the nuclear tRFs are derived from the 5’ half regions of the parental tRNAs. The patterns of tRFs formed during *T. cancriformis* development were investigated in detail.

## Results and discussion

### Small RNAs are derived from mitochondrial and nuclear tRNAs in *T. cancriformis*

As reported recently [23], a deep sequencing analysis of the *T. cancriformis* small RNAs in each of its six developmental stages (Additional file 1: Table S1 and Additional file 1: Figure S1) was performed, and 151,340,419 reads were obtained (Additional file 1: Figure S2). After the low-quality reads were discarded, 1,162,917 unique reads were retained. Three peaks were observed in the size distribution of the small RNA reads in each stage (Additional file 1: Figure S1). The first peak, at approximately 22 nt, corresponded to the mature miRNA fraction, according to a previous study [23, 25]. The second peak, ranging in size from 26 to 28 nt, was similar in size to the piRNAs [26]. It is noteworthy that the third peak, at 32 nt, was specifically detected in the adult stage (Additional file 1: Figure S1). A nucleotide sequence comparison of the 32-nt small RNA reads and predicted tRNA sequences in the nuclear genomic DNA contig sequences revealed that 84.6 % of the 32-nt small RNAs were derived from tRNA^Gly^(GCC). This result indicates that large amounts of tRFs, containing specific regions of the mature parental tRNAs, are expressed in the adult stage of *T. cancriformis*. Therefore, we focused on *T. cancriformis* tRFs, and analyzed them on a large scale. Because approximately half the small transcripts around 22 nt long are miRNAs [23], we focused on the 25–45 nt small RNAs and searched for read sequences that could be mapped to either mitochondrial or nuclear tRNA genes in *T. cancriformis*. We discuss the smaller tRFs of 18–24 nt in a following section.

**Fig. 1:**
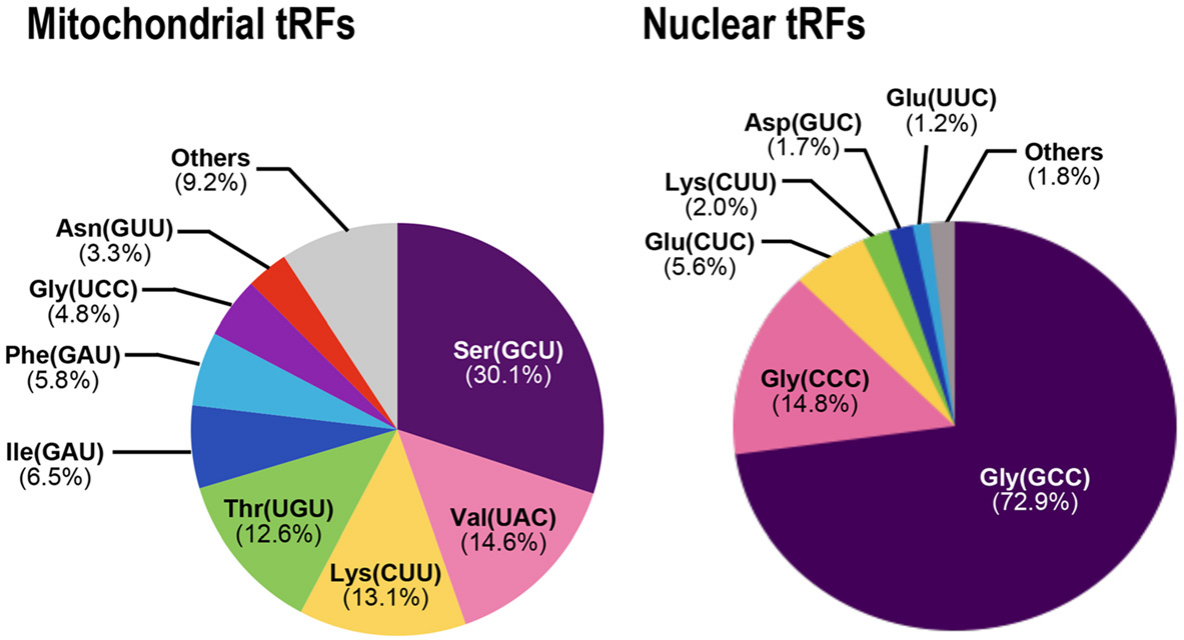
Pie charts summarizing the proportions of mitochondrial and nuclear tRFs in *T. cancriformis.* The tRFs were categorized by their corresponding anticodons

To identify the mitochondrial tRFs, we first predicted all the tRNA genes in the *T. cancriformis* mitochondrial genome sequence [27] with tRNAscan-SE 1.3.1 [28, 29]. In this way, we identified 22 mitochondrial tRNA genes, including seven reannotated tRNA genes, in this study (Additional file 1: Table S2). A comparative sequence analysis then revealed that 15.7 % of the small RNA reads (12,240 reads in all) that mapped to the mitochondrial DNA were derived from mitochondrial tRNAs (Additional file 1: Figure S3). Compared with the number of all small RNA reads, the read count for mitochondrial tRFs was very low (approximately 0.015 %), suggesting the minuscule expression of mitochondrial tRFs. The mitochondrial tRFs were derived from mitochondrial tRNAs corresponding to 16 of the 22 anticodons (Table 1). Among the mitochondrial tRF species detected, tRF^Ser^(GCU) was most abundant (30.1 % of all mitochondrial tRF reads; Fig. 1).

**Table 1:** Summary of the deep sequencing analysis of tRFs in *T. cancriformis*

Isotype	Total read count	Main tRF region
**Mitochondrial tRF**		
Ser (GCU)	3,678	5’ half
Val (UAC)	1,784	3’ end
Lys (CUU)	1,606	5’ half
Thr (UGU)	1,547	5’ end and 3’ half
Ile (GAU)	795	5’ end and 3’ half
Phe (GAA)	713	3’ end
Gly (UCC)	583	AC stem-loop
Asn (GUU)	403	3’ end and AC stem-loop
Met (CAU)	316	AC stem-loop
Tyr (GUA)	294	5’ half and AC stem-loop
Asp (GUC)	218	3’ end and AC stem-loop
Cys (GCA)	144	3’ half
Pro (UGG)	67	5’ and 3’ end
Gln (UUG)	56	5’ half and AC stem-loop
Leu (UAA)	30	3’ end
Ala (UCG)	6	3’ end
**Nuclear tRF**		
Gly (GCC)	3,674,244 ^a,b^	5’ half
Gly (CCC)	749,207	5’ half
Glu (CUC)	280,322 ^c^	5’ half
Lys (CUU)	102,190	5’ half
Asp (GUC)	88,065	5’ half
Glu (UUC)	62,011 ^c^	5’ end
His (GUG)	29,698	5’ end
Thr (UGU)	10,286	3’ end
Gly (UCC)	9,046 ^b^	5’ half
SeC (UCA)	6,911	5’ half
Pro (CGG)	6,616 ^b,c^	5’ half
Pro (UGG)	5,842 ^b,c^	5’ half
Cys (GCA)	3,808	5’ half
Gln (CUG)	3,654 ^b,c^	AC stem-loop
Ala (CGC)	3,413	5’ half
Gln (UUG)	2,478 ^b,c^	5’ half

^a^ All reads that could be mapped to both tRNA and other nuclear genomic regions were removed

^b^ All reads that could be mapped to tRNA genes containing polymorphic site(s) were removed

^c^ All reads that could be mapped to tRNA genes with several different anticodons were removed

To look for nuclear tRFs, the nuclear tRNA genes in the draft *T. cancriformis* nuclear genome were also predicted with tRNAscan-SE. After removing the pseudo-tRNAs, the tRNAs predicted at the end of the contig sequences, and the tRNAs that contained polymeric site(s), at least 254 genes corresponding to 45 anticodons were reliably identified as *T. cancriformis* tRNA genes. We found that the nuclear tRFs identified in this study were derived from nuclear tRNAs corresponding to at least 39 of the 45 anticodons. A further sequence analysis revealed that 6.9 % of the small RNA reads (5,048,874 reads in all) that mapped to the *T. cancriformis* nuclear genome were derived from nuclear tRNAs. The total read number of nuclear tRFs was 412 times larger than the number of mitochondrial tRFs, indicating that the majority of tRFs in the cell are derived from nuclear tRNAs. We noted that a large percentage of nuclear tRFs were derived from nuclear tRNA^Gly^(GCC) (72.9 % of nuclear tRF reads; Fig. 1), and this 32-nt tRF^Gly^(GCC) was the most abundantly detected tRF of all variants.

These results indicate that both mitochondrial and nuclear tRFs are derived from parental tRNAs with specific anticodons. It should be noted that not all parental nuclear tRNA genes corresponding to tRFs were identified in the present analysis. Firstly, the tRF locus could not be determined in all cases because some tRF reads could be mapped to both tRNA genes and other regions of the nuclear genomic DNA. Secondly, the prediction of the nuclear tRNA genes was incomplete because we used the draft genome of *T. cancriformis*. Short tRFs (15–25 nt long) have also been reported in several other organisms and some of these tRFs act as miRNAs [14, 15, 19]. Therefore, the *T. cancriformis* small RNA fraction of around 22 nt may also contain smaller tRFs. However, mapping these short tRFs onto each parental tRNA is technically difficult because tRNA genes occur in large families with highly similar sequences and most nuclear tRNA genes have isoacceptor genes. Furthermore, most *T. cancriformis* tRNAs produce tRFs. To circumvent these problems in this study, we focused on the 25–45 nt tRFs that were completely and reliably mapped to mitochondrial or nuclear tRNA genes.

### Main regions of mitochondrial tRFs in each parental tRNA differ, depending on their anticodons

**Fig. 2:**
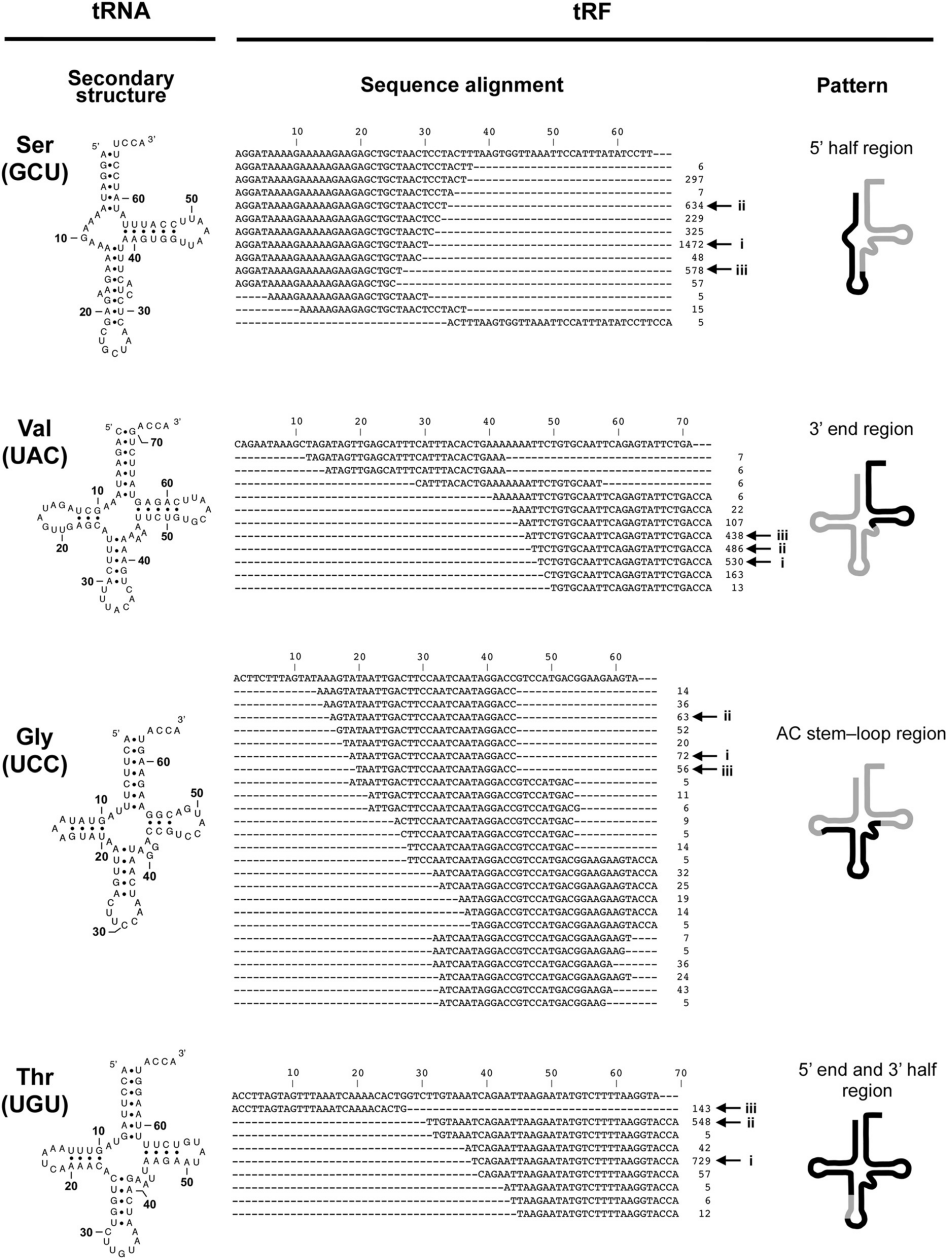
Four examples of mitochondrial tRNAs and their tRFs in *T. cancriformis*. Secondary structures of four mature mitochondrial tRNAs are shown on the left. Nucleotide sequence alignments of each tRNA and its tRFs are shown in the center. Total read counts of each tRF in the six developmental stages are shown (the three most numerous of the tRF variants are indicated with arrows and Roman numerals i–iii). On the right, the main tRF regions in each parental tRNA are shown with a bold line. AC stem–loop region (anticodon stem–loop region). See Additional file 1: Figure S4 for another 12 examples of mitochondrial tRNAs and their tRFs in *T. cancriformi*

The characteristics of mitochondrial tRFs have not been thoroughly investigated until recently, although many nuclear tRFs have been reported in previous studies (see Introduction). To determine the exact positions of the *T. cancriformis* mitochondrial tRFs, all tRF variants (tRFs of different sizes but derived from the same parental tRNA) were aligned with their parental tRNAs. The main regions in the parental tRNAs from which the tRFs were derived differed, depending on the parental anticodon (Table 1). Here, we defined the 5’ or 3’ half tRFs as tRFs cleaved in the anticodon loop region. We defined the 5’ or 3’ end tRFs as tRFs that are shorter than the half tRFs and correspond to either the 5’ or 3’ end of the parental tRNA, respectively. A typical tRF pattern is defined as a representative tRF pattern with higher read numbers than its variants. As exemplified in Fig. 2, a number of tRF^Ser^(GCU) variants were aligned to the 5’ half region of their tRNA, whereas a very small number of tRF^Ser^(GCU) variants were aligned to the 3’ half region of the tRNA. In contrast, tRF^Val^(UAC)s, tRF^Gly^(UCC)s, and tRF^Thr^(UGU)s mapped preferentially to the 3’ end region, the anticodon stem–loop (AC stem–loop) region, and the 5’ end and 3’ half regions, respectively. Another 12 examples of mitochondrial tRFs in *T. cancriformis* are shown in Additional file 1: Figure S4. Overall, the mitochondrial tRFs could be classified into the following patterns of the main tRF regions: 5’ half region, 5’ end region, 3’ half region, 3’ end region, AC stem–loop region, and a mixture of these patterns. A few specific sequences were extremely enriched among the tRF variants. For examples, in the case of tRF^Val^(UAC)s, three specific tRF sequences (Fig. 2, indicated with Roman numerals i–iii) were enriched (81.5 % of all tRF^Val^(UAC)s), suggesting that tRNA cleavage occurred at specific positions in each tRNA, and that mitochondrial tRFs are not random degradation products.

### Altered expression of mitochondrial tRFs during developmental stages

**Fig. 3:**
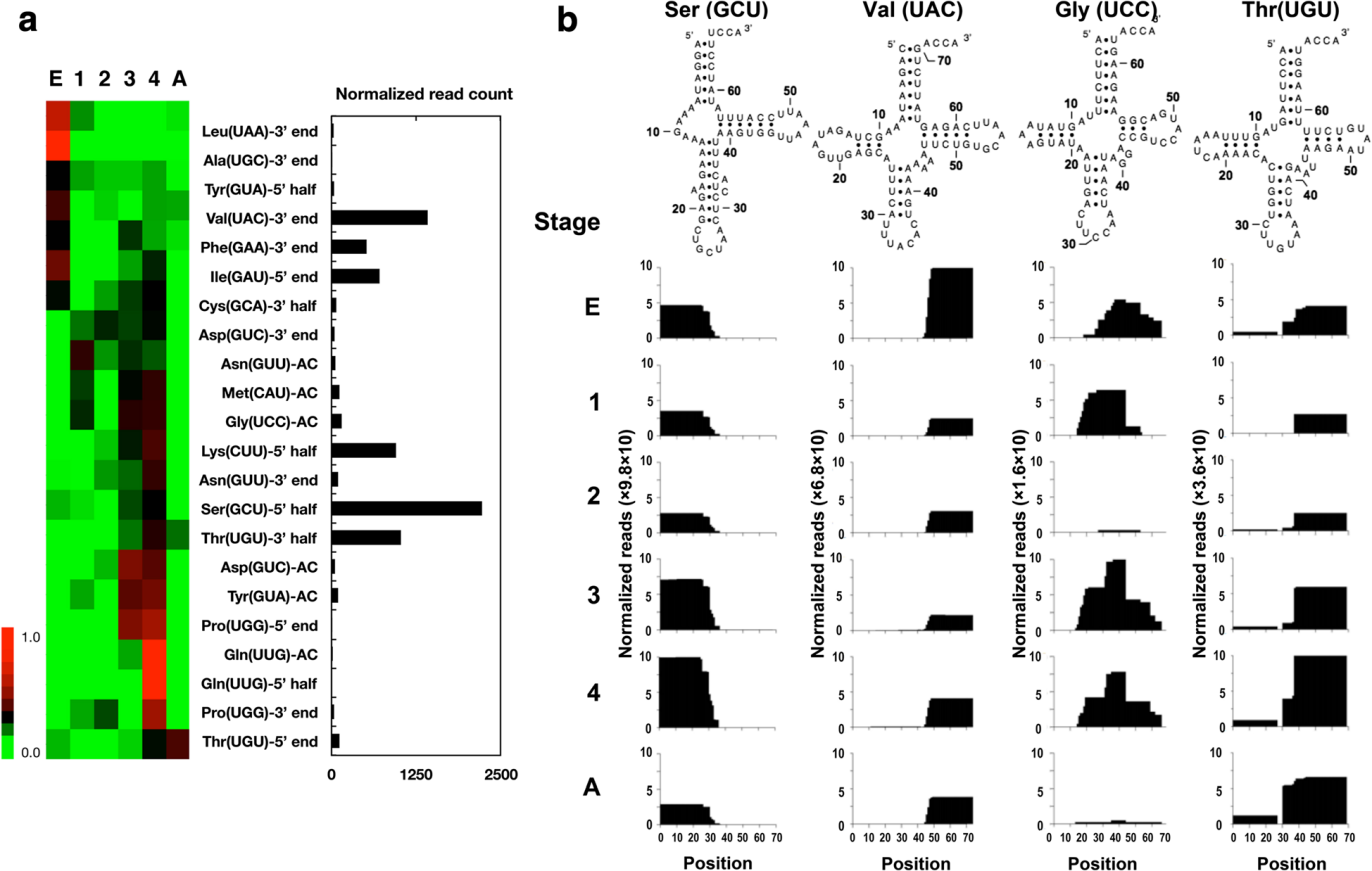
Expression of four mitochondrial tRFs during *T. cancriformis* development. (**a**) Heatmap shows the expression profiles of mitochondrial tRFs during *T. cancriformis* development. Each color in the heatmap represents the relative normalized read counts of the three most-enriched tRFs. Red indicates a high relative read frequency and green indicates a low relative read frequency. (E) Egg; (1) 1st instar larva; (2) 2nd instar larva; (3) 3rd instar larva; (4) 4th instar larva; (**a**) adult. Bar graph shows the total normalized read counts (in all stages) of the three most-enriched tRFs among the tRF variants. (**b**) Accumulation of all tRF reads that mapped to four individual mature mitochondrial tRNA sequences are visualized in each of the six developmental stages (also see Fig. 2 and Additional file 1: Figure S5). Vertical axis indicates the sum of the normalized read counts, and the horizontal axis indicates the base position of each tRNA from the 5’ to 3’ end. “E”, “1–4”, and “A” indicate egg, 1st–4th instar larvae, and adult, respectively

To determine the expression profiles of all mitochondrial tRFs in the developmental stages of *T. cancriformis*, the normalized read counts of the top three tRF variants for each tRNA species were analyzed in the individual developmental stages (Fig. 3a). The expression of the mitochondrial tRFs changed during the developmental stages. The majority of mitochondrial tRFs were highly expressed in the egg or late larval stages (3rd and 4th instar larvae). To understand the expression and positions of all the tRFs in each developmental stage, the accumulation of all the tRF reads mapped to individual mature mitochondrial tRNA sequences was visualized (Fig. 3b and Additional file 1: Figure S5). Half the tRFs were derived from almost the same positions in the parental tRNAs in every stage. For instance, tRF^Ser^(GCU)s and tRF^Val^(UAC)s were derived from the 5’ half region and the 3’ end region of their tRNAs, respectively, throughout all stages. In the case of tRF^Gly^(UCC)s, the positions of the tRFs and their expression changed during development; the tRFs were derived from the 3’ regions and AC stem–loop regions in the egg and 1st instar larval stages, respectively. However, in the 2nd instar larval stage, the expression of both tRFs was very low, but increased again in the 3rd and 4th instar larval stages. The complex production of these mitochondrial tRFs was supported by other mitochondrial tRNAs corresponding to Met(CAU), Tyr(GUA), Gln(UUG), Asp(GUC) and Asn(GUU) (Additional file 1: Figure S5). We surmised that individual tRFs derived from the same tRNAs were expressed at different stages, and that mitochondrial tRNA cleavage is regulated to generate these different tRFs in different developmental stages.

### Large numbers of nuclear tRFs are derived from the 5’ half regions of their tRNAs

**Fig. 4:**
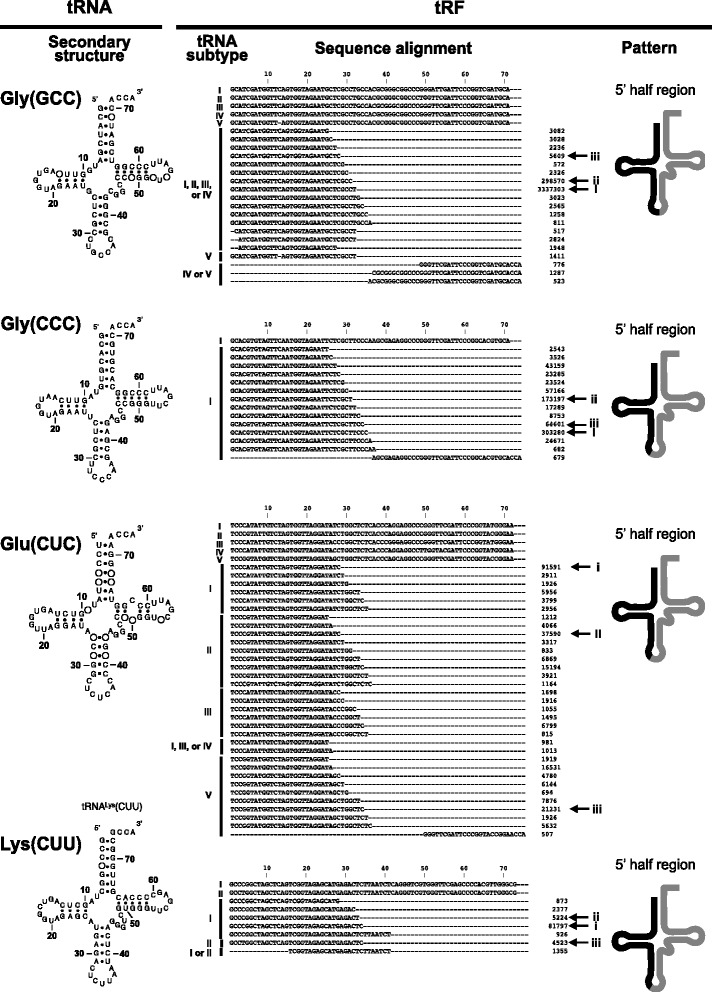
Four examples of nuclear tRNAs and their tRFs in *T. cancriformis*. Examples of highly abundant nuclear tRFs were selected. The secondary structures of the four mature nuclear tRNAs and the nucleotide sequence alignments between these tRNAs and their tRFs are shown (also see Fig. 2 legend). Different tRNA genes but with the same anticodon sequences (tRNA gene subtypes) are shown with upper-case Roman characters (I–V). The white circle in the secondary structure indicates the nonconserved nucleotides among the tRNA subtypes. Highly redundant tRF reads (≥500 reads) were used for the sequence alignments. Also see Additional file 1: Figure S6 for another 12 examples of nuclear tRNAs and their tRFs in *T. cancriformis*

To identify the positions of the *T. cancriformis* nuclear tRFs in their parental nuclear tRNAs, the same analysis was conducted as was applied to the mitochondrial tRFs. The 16 most highly expressed nuclear tRFs were selected and analyzed. Twelve of the 16 nuclear tRFs were derived from the 5’ half regions of their parental tRNAs (Fig. 4 and Additional file 1: Figure S6). Two tRFs for glycine, tRF^Gly^(GCC)s and tRF^Gly^(CCC)s, are members of the 5’ half tRFs and the read counts of these two tRFs accounted for 72.9 % and 14.8 %, respectively, of all the 39 nuclear tRFs (Fig. 1 & Table 1). Some parental tRNA molecules share the same anticodon but differ in their nucleotide sequences, and are called ‘tRNA isodecoders.’ We refer to them as ‘RNA subtypes’ in this paper. Several tRFs belonging to different parental tRNA subtypes corresponded to similar positions, and others corresponded to different positions on the parental tRNA, depending on the subtype (Fig. 4 and Additional file 1: Figure S6). For instance, tRF^Glu^(CUC)s were derived from five parental tRNA^Glu^(CUC) subtypes (I–V) consisting of 33 variants, and 32 of them were derived from the 5’ region of the parental tRNA (Fig. 4). Among these tRF^Glu^(CUC) variants, the read counts of two 28-nt tRFs (91,591 reads and 37,590 reads) accounted for approximately half the reads of the 33 tRF^Glu^(CUC) variants (indicated as (i) and (ii) in the tRF^Glu^(CUC)s in Fig. 4), suggesting that the 28-nt tRFs are preferentially produced from the 5’ regions of tRNA^Glu^(CUC)s. tRF^Asp^(GUC)s are examples of tRFs correspond to different positions according to the parental subtype (Additional file 1: Figure S6). tRNA^Asp^(GUC) has five subtypes (I–V) consisting of 18 variants, and tRF^Asp^(GUC)s derived from subtypes I–III of tRNA^Asp^(GUC) were detected in this analysis. Among these tRF^Asp^(GUC)s, the variants derived from subtypes I–III corresponded to the 5’ region of tRNA^Asp^(GUC), and the variants derived from subtype III corresponded to the 3’ region of tRNA^Asp^(GUC).

### Expression of nuclear tRFs in *T. cancriformis* developmental stages

**Fig. 5:**
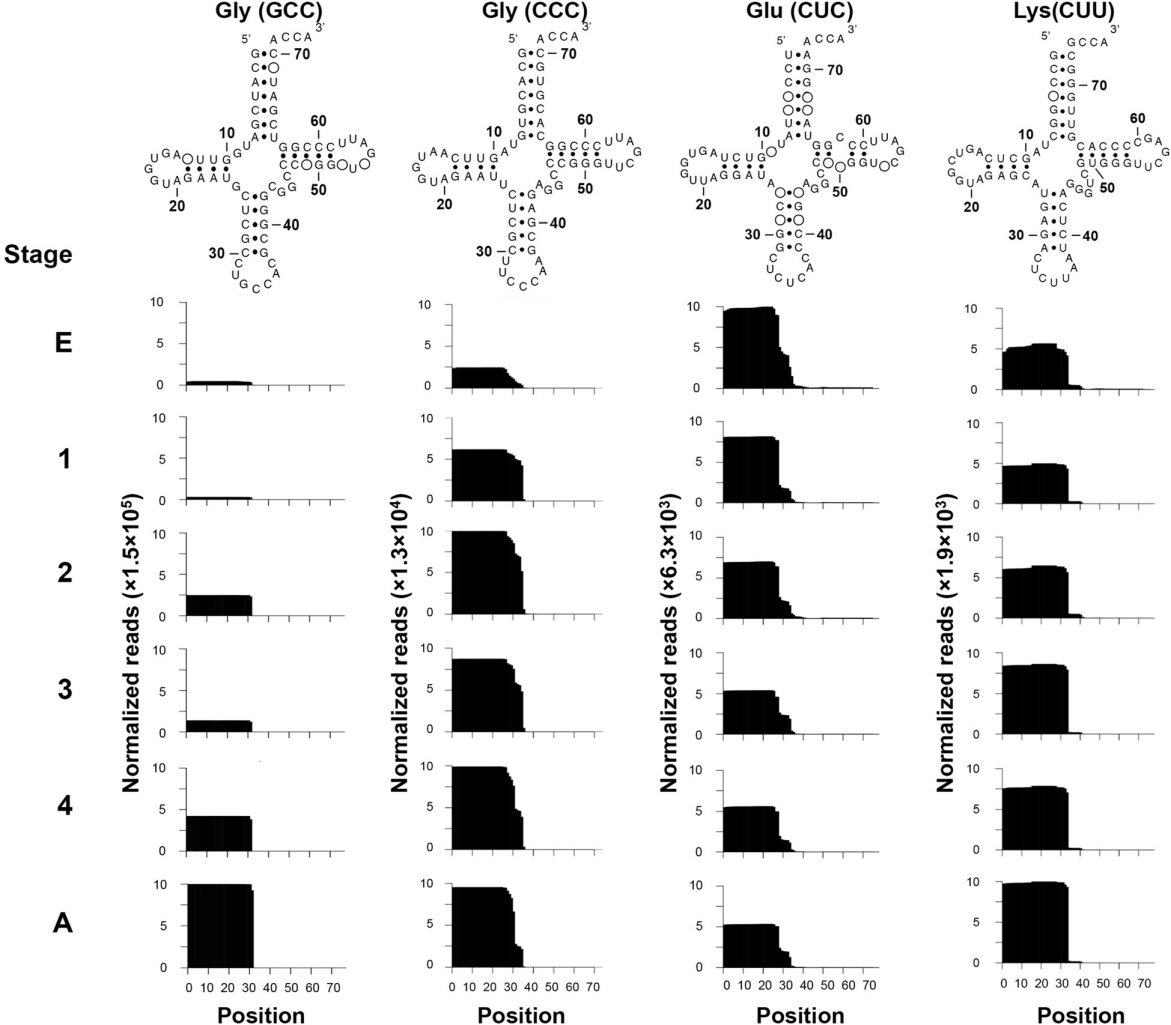
Expression of four nuclear tRFs during *T. cancriformis* development. The accumulation of all tRF reads that mapped to four individual mature nuclear tRNA sequences are visualized in each of the six developmental stages, as in Fig. 3B (also see Fig. 4 and Additional file 1: Figure S7)

The expression of 16 nuclear tRFs with high read numbers was also analyzed during the six developmental stages. Each of the 16 nuclear tRFs was usually derived from the same position in its parental tRNA during *T. cancriformis* development (Fig. 5 and Additional file 1: Figure S7). The changes in expression during development can be roughly classified into the following three patterns: (1) expression increased in accordance with the developmental stage (tRF^Gly^(GCC), tRF^Gly^(CCC), tRF^Lys^(CUU), tRF^Sec^(UCA)); (2) expression decreased in accordance with the developmental stage (tRF^Glu^(CUC), tRF^Asp^(GUC), tRF^His^(GUG)); and (3) highly expressed in the egg (tRF^Gly^(UCC), tRF^Pro^(UGG), tRF^Cys^(GCA), tRF^Gln^(UUG), tRF^Thr^(UGU), tRF^Gln^(CUG)). These results show that the expression of the nuclear tRFs changes during development, as does the expression of mitochondrial tRFs. However, their fragmentation patterns are rather simple and most nuclear tRFs are derived from the 5’ half regions of the parental tRNAs.

**Fig. 6:**
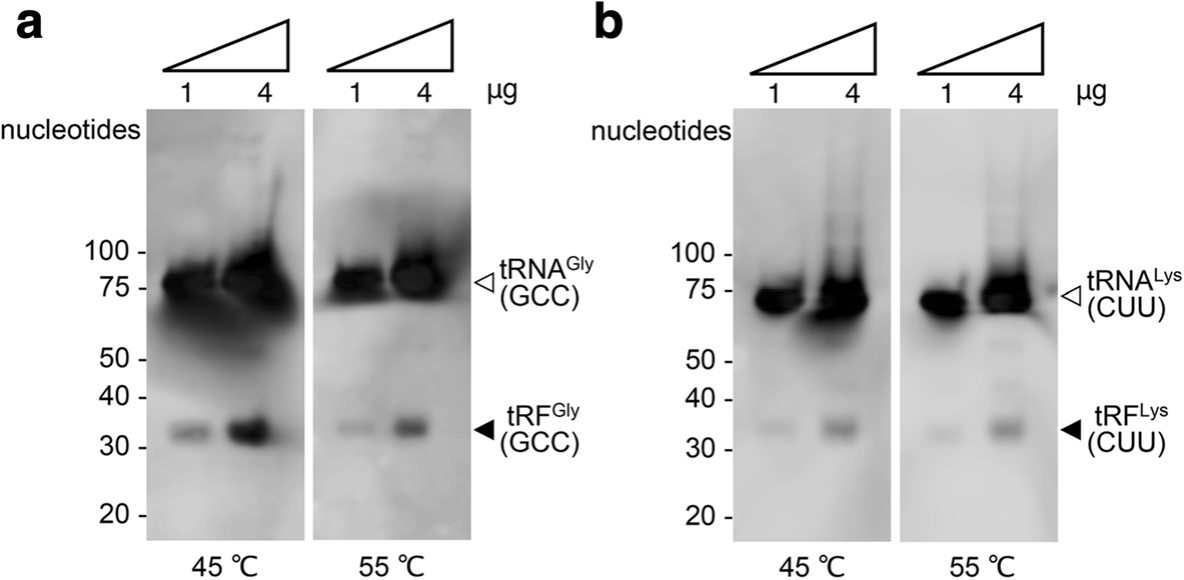
Northern blot analysis of two *T. cancriformis* nuclear tRFs. Expression of (**a**) nuclear tRF^Gly^(GCC) and (**b**) nuclear tRF^Lys^(CUU) was detected in the adult stage of *T. cancriformis* with a northern blotting analysis using probes specific to the most-enriched tRF for each anticodon. Total RNA (1 or 4 μg) isolated from the adult stage was used. Washing treatments were performed at either 45 or 55 °C

To confirm the expressions of the nuclear tRFs in *T. cancriformis*, a northern blotting analysis was conducted in the adult stage, using oligonucleotides specific for two highly expressed tRFs, tRF^Gly^(GCC)s and tRF^Lys^(CUU)s. The main mature parental tRNA bands were clearly detected (approximately 75 nt) and their tRFs were detected at around 30–35 nt in each case (Fig. 6). The sizes of the tRFs were consistent with those of the abundant and corresponding reads in the deep sequencing analysis (Fig. 4). The northern analysis also suggested that the tRFs correspond to only a small proportion of the mature parental tRNAs in each case. Therefore, although several variant reads were detected in the deep sequencing analysis, it was difficult to distinguish and visualize all these variants in the northern blotting analysis, probably because the read numbers varied so much among the tRF variants, and it is difficult for shorter and less abundant bands to form stable hybrids and thus produce strong signals. It was also difficult to obtain clear mitochondrial tRF signals with the northern blotting analysis because their expression is low. We conducted several northern blot analyses of these tRFs. However, the 20–30-nt antisense oligonucleotides were not specific to many tRFs and cross-hybridized with other highly homologous tRFs. Therefore, for now, the only way to accurately estimate the amounts of both nuclear and mitochondrial tRFs is with a deep sequencing (RNA-seq) analysis.

### Perspective on *T. cancriformis* tRFs

In this study, we identified tRFs derived from parental tRNAs encoded in both the nuclear and mitochondrial genomes of *T. cancriformis*, and determined the dynamics of their expression during development. Recently, there have been many reports of the nuclear tRFs in various organisms. However, the number of publications about mitochondrial tRFs is still limited. It has been reported that in *Tetrahymena thermophile*, mitochondrial tRF^Phe^(GAA) is generated by the starvation-induced cleavage of the tRNA anticodon loop [13]. Analyses of the biological functions of tRFs have predominantly investigated nuclear tRFs. For example, an angiogenin-induced 5’ tRF inhibits the initiation of translation in human cells [10]. A *Tetrahymena* Piwi protein bound to a 3’ tRF activates the exonuclease Xrn2 for pre-rRNA processing [30]. It has also recently been shown that tRF^Tyr^ is generated in CLP1 kinase-dead mice, defective in pre-tRNA splicing, and the accumulated tRF^Tyr^ induced a loss of motor neurons in the mice [31]. Thus, the functions of tRFs may vary, but are related to some cellular stress response.

Although a typical tRNA molecule has a cloverleaf-like secondary structure, some mitochondrial tRNAs reportedly lack either the D- or T-arm [32]. The armless tRNAs (which structurally resemble tRNA halves) are found in the mitochondrial genomes of several species [33–35] and at least some of them are actually expressed, processed, and aminoacylated [34, 36]. These findings suggest that the whole cloverleaf-like structure is not required for the basic translational functions of tRNAs. Di Giulio et al. hypothesized that the ancestral tRNA was encoded by two separate minigenes, which later fused to encode the modern tRNAs [37–39]. The recent discovery of split tRNA genes, in which two or three segments of the tRNA are merged to form the mature tRNA [3–5], may support this hypothesis [11, 40]. Randau and Soll suggested that split tRNA genes arose by gene division during the process of genomic rearrangement, and have since been maintained as a protective mechanism against the integration of mobile genetic elements [41]. In contrast, Seligmann proposed a unique theory, the pocketknife tRNA hypothesis, in which the sidearms that form the tRNA fragments corresponding to the tRNA halves function in translation [42, 43]. However, there is not yet any direct evidence that tRFs are involved in translation as adapter molecules that link the nucleotide sequences of mRNAs to the amino acid sequences of proteins. Further research is required to extend and consolidate this view.

In our analysis, the *T. cancriformis* tRFs were derived from specific regions of the parental tRNAs, suggesting that these tRFs are not random products of tRNA degradation but are functional molecules. However, it is also true that there is no exact criterion by which to distinguish functional tRFs and degradation products in the current analysis. We predicted the *T. cancriformis* Argonaute/Piwi proteins in our previous study [23], and the tRFs might bind to one of these proteins. However, important questions are still unresolved. Why are most nuclear tRFs derived from the 5’ regions of the parental tRNAs? Why are huge numbers of tRF variants generated and what enzyme is responsible? To answer these questions and to identify the biological function(s) of tRFs in *T. cancriformis*, it will be important, as the next step, to identify the tissues or cells in which each specific tRF is generated.

## Conclusions

(i) A deep sequencing analysis revealed that the *T. cancriformis* tRFs are derived from mitochondrial and nuclear tRNAs corresponding to 16 and 39 anticodons, respectively.
(ii) The total read number of nuclear tRFs is approximately 400 times larger than the read number of mitochondrial tRFs.
(iii) The main regions in the parental tRNAs from which the mitochondrial tRFs are derived differ greatly, and depend on the parental anticodon. However, most nuclear tRFs are derived from the 5’ half regions of the parental tRNAs.
(iv) Some tRFs correspond to different positions according to the parental subtype.
(v) The expression of mitochondrial and nuclear tRFs differs during the six stages of *T. cancriformis* (egg, 1st–4th instars, and adult).
(vi) The small RNA fraction around 22 nt may also contain smaller tRFs, whose parental tRNAs have not been identified.

## Methods

### *Triops cancriformis* culture

*Triops cancriformis* (adults and eggs) were obtained from two rice fields (at Sakata and Higashitagawa-gun, in Yamagata, Japan). *Triops cancriformis* was cultured as reported previously [23].

### Prediction of mitochondrial and nuclear tRNA genes

The mitochondrial DNA sequence of *T. cancriformis* (accession number NC004465) [27] was downloaded from the GenBank sequence database at the National Center for Biotechnology Information (NCBI). The mitochondrial tRNA genes were predicted with tRNAscan-SE version 1.3.1 [28, 29] using the organellar (mitochondrial/chloroplast) tRNA search option, and some tRNA genes were reannotated in this study (see Additional file 1: Table S2).

The draft nuclear genome sequence of the organism was used to predict the nuclear tRNA genes of *T. cancriformis*. Nuclear tRNA genes were predicted with tRNAscan-SE version 1.3.1 using both the eukaryotic and bacterial tRNA search options. The Cove scores (bit scores) calculated with tRNAscan-SE for each tRNA were obtained at the same time. We defined *T. cancriformis* tRNA genes as those genes with higher COVE scores on the eukaryotic search option than on the bacterial tRNA search option.

### Computational extraction of mitochondrial and nuclear tRFs

To extract reliable small RNA reads, the following four filtering steps were performed. First, reads containing sequence errors (N) and low-quality reads (PHRED quality scores < 20) were discarded from the raw deep sequencing data for the small RNAs (Additional file 1: Figure S2, step 1). Reads that were sequenced fewer than five times were also removed (Additional file 1: Figure S2, step 1). Only small RNA reads of 25–45 nt were used for further analysis (Additional file 1: Figure S2, step 2). Using a BLAST alignment [44], the 25–45 nt reads were then mapped to the mitochondrial DNA sequence (Additional file 1: Figure S2, step 3). Reads that mapped perfectly to mitochondrial tRNA genes were defined as mitochondrial tRFs. It is well known that the CCA tails are added to the 3’ ends of tRNAs during their maturation, so the 3’ CCA tails are not encoded in the mitochondrial or nuclear DNA sequences in eukaryotes [45]. Therefore, the CCA tails were masked, and the reads without the CCA tail sequences were mapped to the mitochondrial DNA sequence. If the CCA-tail-masked reads mapped perfectly to regions of the mitochondrial tRNA genes, these reads were defined as mature-mitochondrial-tRNA-derived fragments.

Reads that could not be mapped to the mitochondrial DNA sequence were mapped to the nuclear DNA contig sequences using a BLAST alignment (Additional file 1: Figure S2, step 4). Nuclear tRFs were defined as reads that aligned perfectly to a nuclear parental tRNA. The nuclear tRFs were also mapped using the approach used to extract the mitochondrial tRFs with CCA tails. All reads that mapped to (a) both tRNA and other nuclear regions, (b) tRNA genes containing polymorphic site(s), and (c) tRNA genes with several different anticodons were removed from the tRF reads (see Table 1).

### Expression pattern analysis of tRFs during *T. cancriformis* development

To compare the expression levels of tRFs in the six different developmental stages of *T. cancriformis*, the tRF reads were normalized using two small RNA spikes [23]. The expression profiles of the mitochondrial tRFs were clustered with Cluster 3.0 [46], and visualized with Java Treeview [47].

### Northern blot analysis

Total RNA was isolated from *T. cancriformis* (adult stage) using TRIzol Reagent (Invitrogen, Carlsbad, CA, USA), according to the manufacturer’s protocol. The total RNA (1 or 4 μg) was separated with denaturing (urea) polyacrylamide gel electrophoresis, and electrotransferred to Hybond-N+ membrane (GE Healthcare, Piscataway, NJ, USA). The blots were UV cross-linked and prehybridized with buffer containing 0.5 % SDS, 4 × SSC, and 50 × Denhardt’s solution for 30 min at 45 °C. The antisense oligonucleotide probes were labeled with the Biotin 3’ End DNA Labeling Kit (Pierce Biotechnology, Rockford, IL, USA). The blots were then hybridized with the 3’-labeled antisense oligonucleotide in the same buffer overnight at 45 °C. The membranes were washed with buffer containing 0.1 % SDS and 0.2 × SSC at 45 or 55 °C. The nonisotopic blots were visualized with ECF Substrate (GE Healthcare) and the images recorded with Molecular Imager FX Pro (Bio-Rad Laboratories, Hercules, CA, USA). The following oligonucleotide probes were used:

Probe A, 5’-AGGCGAGCATTCTACCACTGAACCATCGATGC-3’ to detect 5’ half tRF^Gly^(GCC).

Probe B, 5’-GAGTCTCATGCTCTACCGACTGAGCTAGCCGGGC-3’ to detect 5’ half tRF^Lys^(CUU).

### Availability of supporting data

The deep sequencing data for *T. cancriformis* small RNAs in each of the six developmental stages (egg, 1st–4th instar larvae, and adult) and a draft nuclear genome [23] were used. The nucleotide sequences of *T. cancriformis* small RNAs and nuclear DNA have been deposited in the DNA Data Bank of Japan (DDBJ) (http://www.ddbj.nig.ac.jp/index-e.html) under accession numbers PRJDB1672 and PRJDB1662, respectively.

## Additional file

Additional file 1: Tables S1 to S2 and Figures S1 to S7.
**Table S1.** Summary of the small RNA read counts in each of the six developmental stages of *T. cancriformis.* **Table S2.** Predicted tRNA genes in the *T. cancriformis* mitochondrial genome. **Figure S1.** Deep sequencing analysis of small RNAs during *T. cancriformis* development*.* **Figure S2.** Bioinformatic analysis of the *T. cancriformis* small RNAs. **Figure S3.** Proportions of *T. cancriformis* mitochondrial small RNAs (25–45 nt). **Figure S4.** Another 12 examples of mitochondrial tRNAs and their tRFs in *T. cancriformis* (see Fig. 2). **Figure S5.** Expression of another 12 mitochondrial tRFs during *T. cancriformis* development. **Figure S6.** Another 12 examples of nuclear tRNAs and their tRFs in *T. cancriformis*. **Figure S7.** Expression of another 12 nuclear tRFs during *T. cancriformis* development (see Fig. 5).

## Abbreviations

AC: Anticodon
DDBJ: DNA Data Bank of Japan
miRNA: microRNA
mRNA: messenger RNA
NCBI: National Center for Biotechnology Information
tRF: tRNA-derived fragment
tRNA: transfer RNA
