# Supplementary material for: Precise mapping and dynamics of tRNA-derived fragments (tRFs) in the development of Triops cancriformis (tadpole shrimp)
Source: BMC Genet. 2015 Jul 14;16:83. doi: 10.1186/s12863-015-0245-5 (PMC4501094; doi:10.1186/s12863-015-0245-5)
Supplement: Additional file 1: Tables S1 to S2 and Figures S1 to S7. — Table S1. Summary of the small RNA read counts in each of the six developmental stages of T. cancriformis. Table S2. Predicted tRNA genes in the T. cancriformis mitochondrial genome. Figure S1. Deep sequencing analysis of small RNAs during T. cancriformis development. Figure S2. Bioinformatic analysis of the T. cancriformis small RNAs. Figure S3. Proportions of T. cancriformis mitochondrial small RNAs (25–45 nt). Figure S4. Another 12 examples of mitochondrial tRNAs and their tRFs in T. cancriformis (see Fig. 2). Figure S5. Expression of another 12 mitochondrial tRFs during T. cancriformis development. Figure S6. Another 12 examples of nuclear tRNAs and their tRFs in T. cancriformis. Figure S7. Expression of another 12 nuclear tRFs during T. cancriformis development (see Fig. 5). [file 12863_2015_245_MOESM1_ESM.pdf]

Additional File 1:

Precise mapping and dynamics of tRNA-derived fragments (tRFs) in the development of *Triops cancriformis* (tadpole shrimp)

Yuka Hirose[1,2], Kahori T. Ikeda[1,2], Emiko Noro[1], Kiriko Hiraoka[1], Masaru Tomita[1,2,3], and Akio Kanai[1,2,3]

1. Institute for Advanced Biosciences, Keio University, Tsuruoka 997-0017, Japan
2. Systems Biology Program, Graduate School of Media and Governance, Keio University, Fujisawa 252-8520, Japan
3. Faculty of Environment and Information Studies, Keio University, Fujisawa 252-0882, Japan

*Corresponding Author: Akio Kanai, Ph.D.

Table S1. Summary of the small RNA read counts in each of the six developmental stages of *T. cancriformis*

	Egg		1st instar		2nd instar		3rd instar		4th instar		Adult	
	**Total reads**	**(Unique reads)**	**Total reads**	**(Unique reads)**	**Total reads**	**(Unique reads)**	**Total reads**	**(Unique reads)**	**Total reads**	**(Unique reads)**	**Total reads**	**(Unique reads)**
**Raw reads**	30,129,430	(5,633,241)	19,451,182	(3,527,456)	25,213,783	(4,041,614)	22,727,822	(3,857,810)	26,237,396	(3,695,978)	27,580,806	(3,943,449)
**Step 1 (Extraction of reliable reads)**	23,438,746	(579,881)	15,019,413	(447,023)	20,078,802	(536,984)	17,921,148	(455,710)	21,597,504	(462,298)	22,625,712	(476,686)
**Step 2 (Extraction of 25–45-nt reads)**	12,881,697	(416,663)	11,185,290	(372,367)	14,544,648	(446,522)	11,979,332	(382,903)	13,977,767	(386,062)	15,825,698	(410,439)
**Total**	80,394,432 (900,174)											

Table S2. Predicted tRNA genes in the *T. cancriformis* mitochondrial genome

tRNA gene	Strand	Start position	End position	Length (nt)
Ile(GAU)	+	1	65	65
Gln(UUG)	-	131	63	69
Met(CAU)	+	128	192	65
Trp(UCA)	+	1,188	1,257	70
Cys(GCA)*	-	1,310	1,248	63
Tyr(GUA)*	-	1,382	1,320	63
Leu(UAA)	+	2,915	2,982	68
Lys(CUU)	+	3,669	3,738	70
Asp(GUC)*	+	3,740	3,803	64
Gly(UCC)*	+	5,414	5,476	63
Ala(UGC)*	+	5,829	5,895	67
Arg(UCG)	+	5,901	5,964	64
Asn(GUU)	+	5,967	6,036	70
Ser(GCU)	+	6,037	6,101	65
Glu(UUC)	+	6,102	6,167	66
Phe(GAA)	-	6,233	6,169	65
His(GUG)	-	8,025	7,959	67
Thr(UGU)*	+	9,657	9,722	66
Pro(UGG)	-	9,786	9,723	64
Ser(UGA)*	+	11,421	11,491	71
Leu(UAG)	-	12,500	12,436	65
Val(UAC)	-	13,877	13,807	71

* Positions of these tRNA genes are reannotated in this study

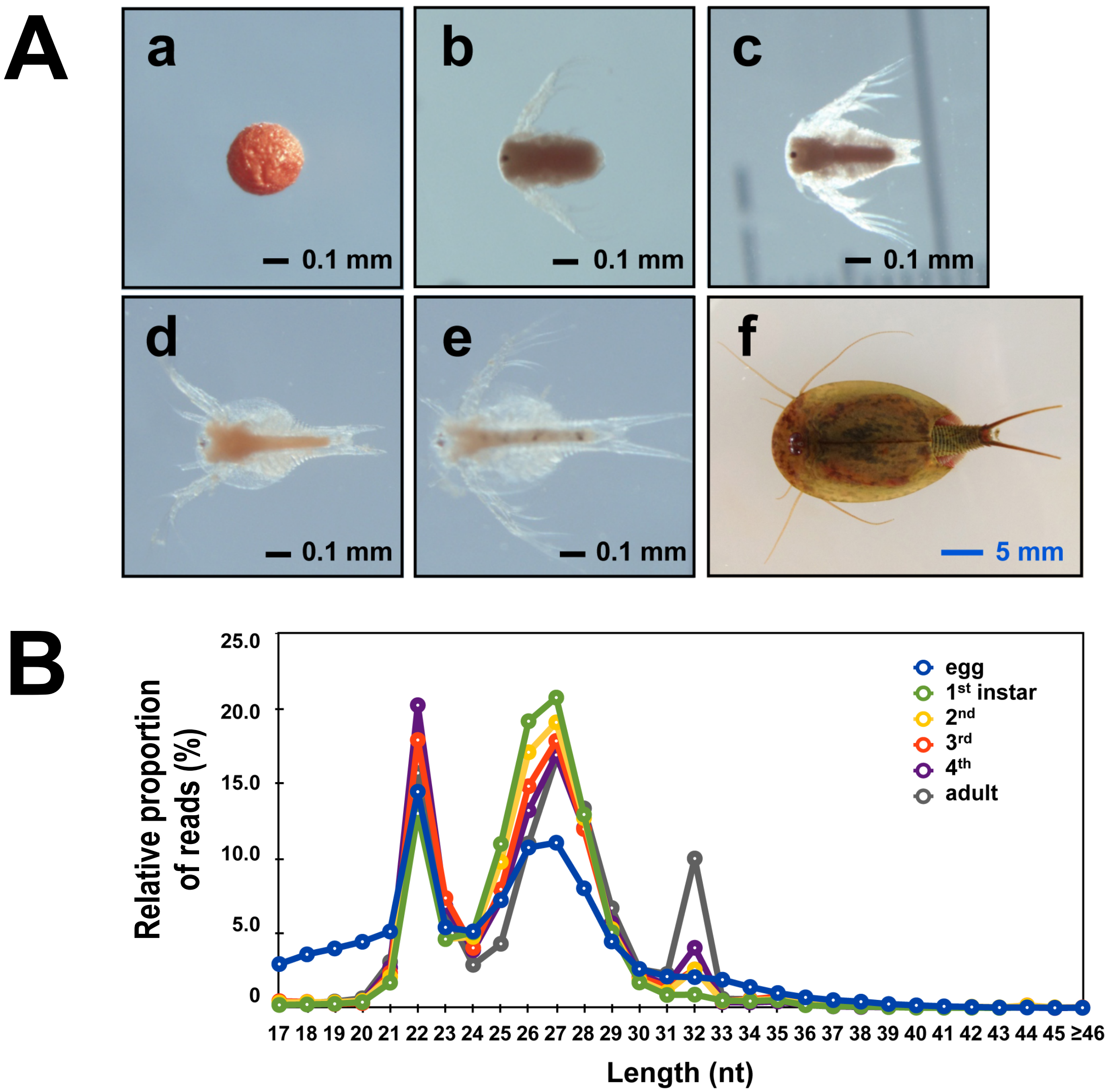

Figure S1. Deep sequencing analysis of small RNAs during *T. cancriformis* development. (A) Morphological changes during *T. cancriformis* development. Scale bars represent 0.5 mm for the egg and larvae of the 1st–4th instars (black bars), and 5 mm for the adult (blue bar). a. Egg; b. 1st instar larva; c. 2nd instar larva; d. 3rd instar larva; e. 4th instar larva; f. adult. (B) Relative numbers of reads based on small RNA lengths. The *y*-axis represents the proportions of small RNA reads (100% represents the total small RNA reads in each stage) [23]. The colors show each stage as indicated in the figure.

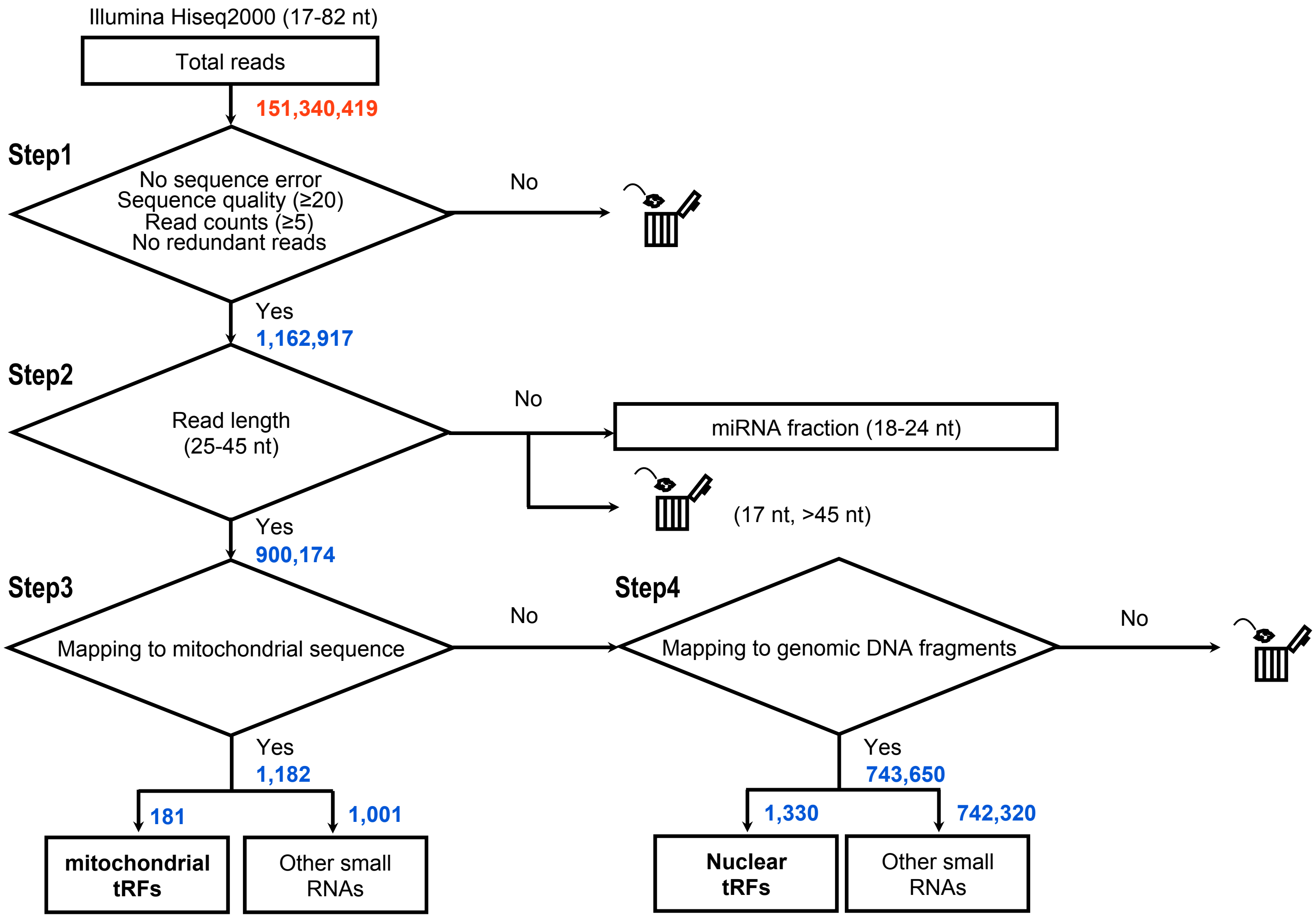

Figure S2. Bioinformatic analysis of the *T. cancriformis* small RNAs. Workflow of the bioinformatic analysis used to extract mitochondrial and nuclear tRFs is shown, and comprises the following four steps: extraction of reliable reads (no sequence errors [N], PHRED quality scores ≥ 20, and read counts ≥ 5) and removal of redundant reads (step 1); extraction of small RNA reads of 25–45 nt (step 2); mapping to the mitochondrial DNA sequence (step 3); and mapping to the nuclear DNA contig sequences (step 4). In steps 3 and 4, the small RNAs were divided into two categories: mitochondrial/nuclear tRFs and other small RNAs. Numbers in red represent the total raw read counts obtained with Illumina HiSeq 2000, and numbers in blue represent the unique read counts obtained in each screening step.

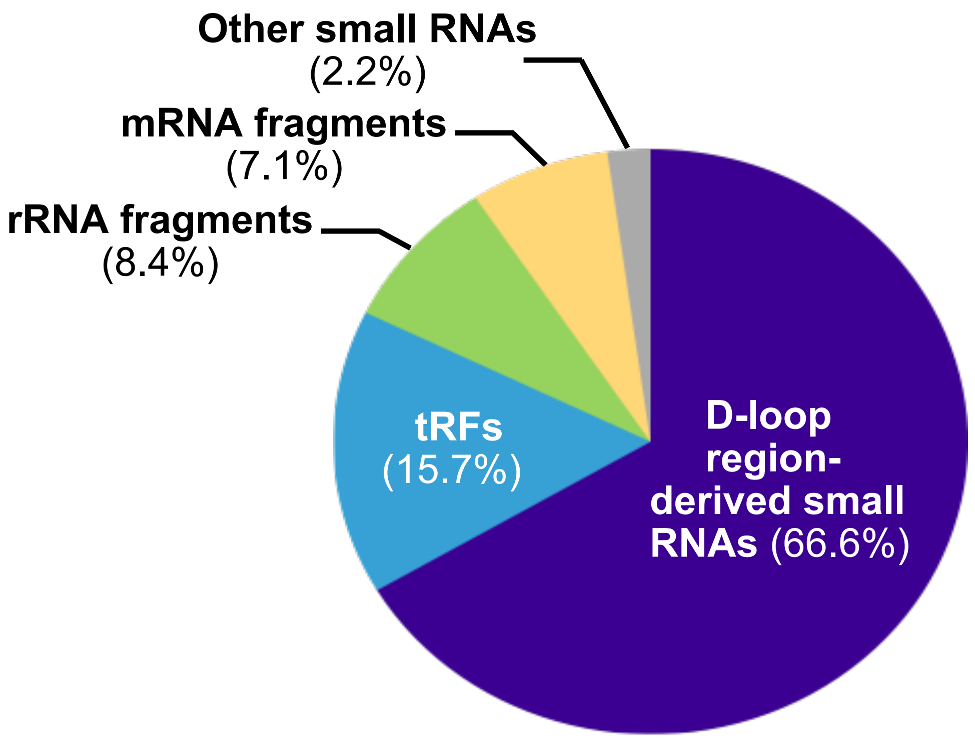

Figure S3. Proportions of *T. cancriformis* mitochondrial small RNAs (25–45 nt). Pie chart shows the proportions of small RNA species that were mapped to the mitochondrial genomic DNA. The small RNAs are categorized according to the regions to which they mapped.

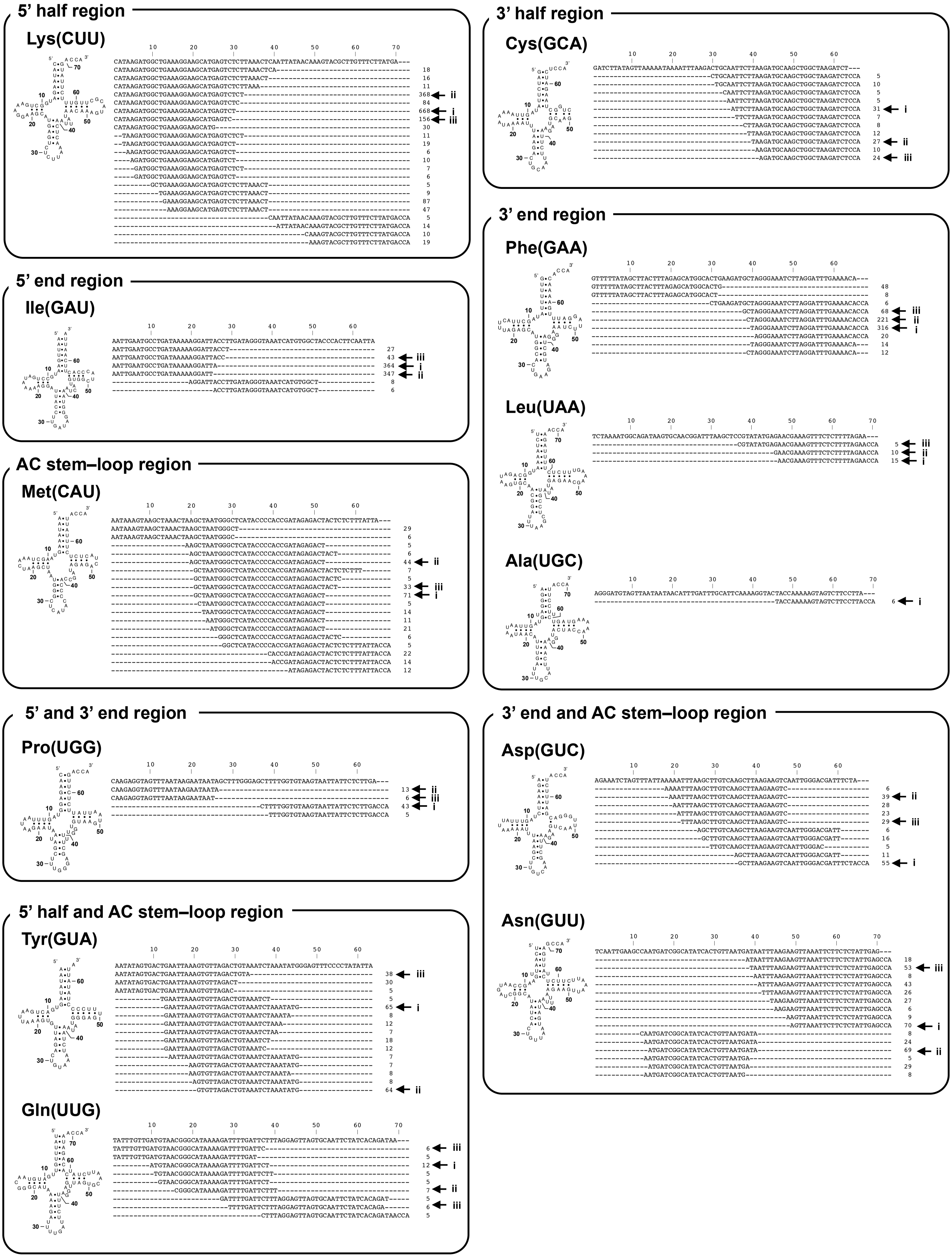

Figure S4. Another 12 examples of mitochondrial tRNAs and their tRFs in *T. cancriformis* (see Fig. 2). Titles in boxes represent the main tRF regions in each parental tRNA. Secondary structure of the mature mitochondrial tRNA is shown on the left. Nucleotide sequence alignment between the tRNA and its tRFs is shown on the right. Total read counts for each tRF in the six developmental stages are also shown. Top three read counts among the tRF variants are indicated with arrows and Roman numerals i–iii.

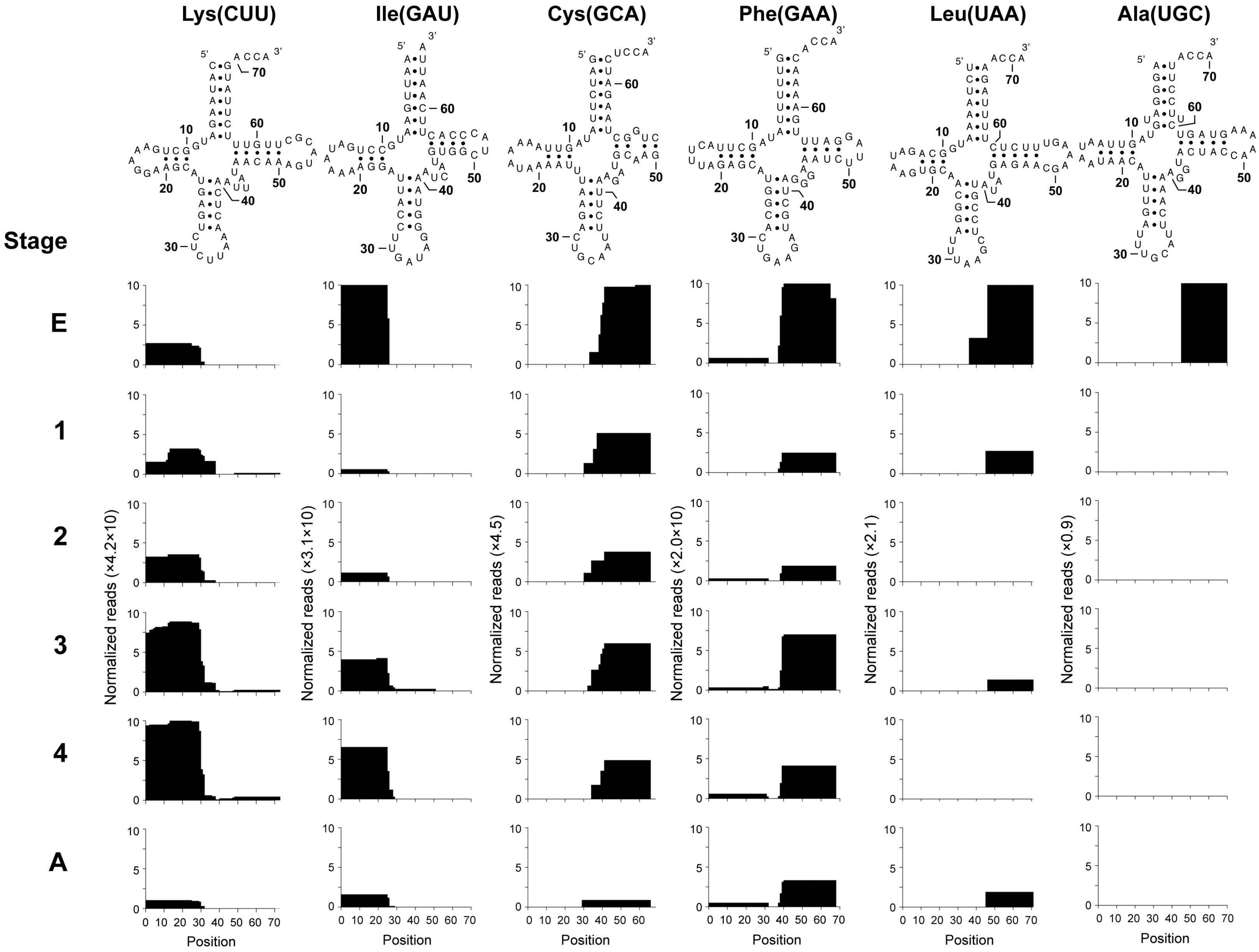

Figure S5. Expression of another 12 mitochondrial tRFs during *T. cancriformis* development. The accumulations of all tRF reads that were mapped to 12 individual parental mitochondrial tRNA sequences in each of the six developmental stages are represented graphically (see Fig. 3).

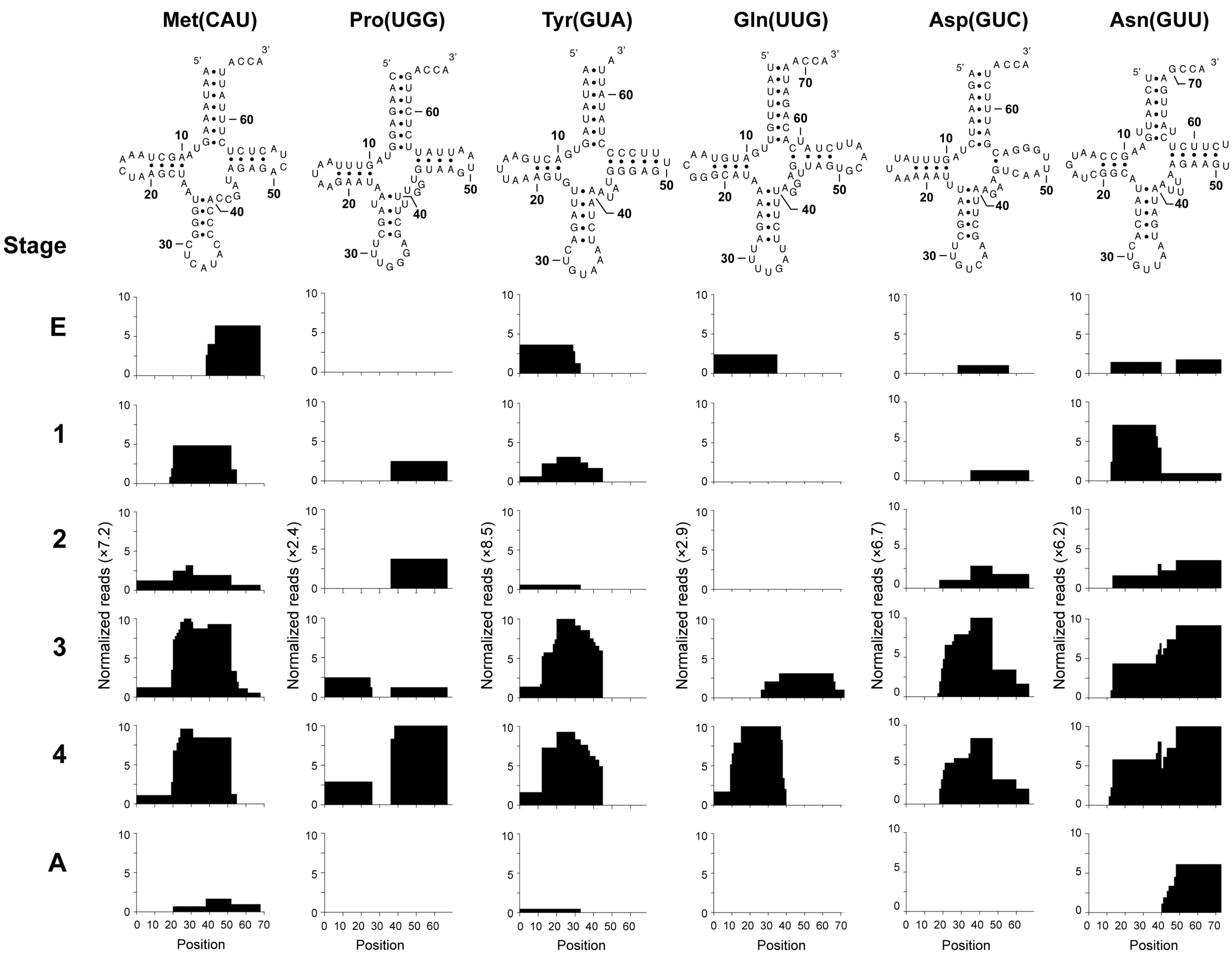

Figure S5 (Continued)

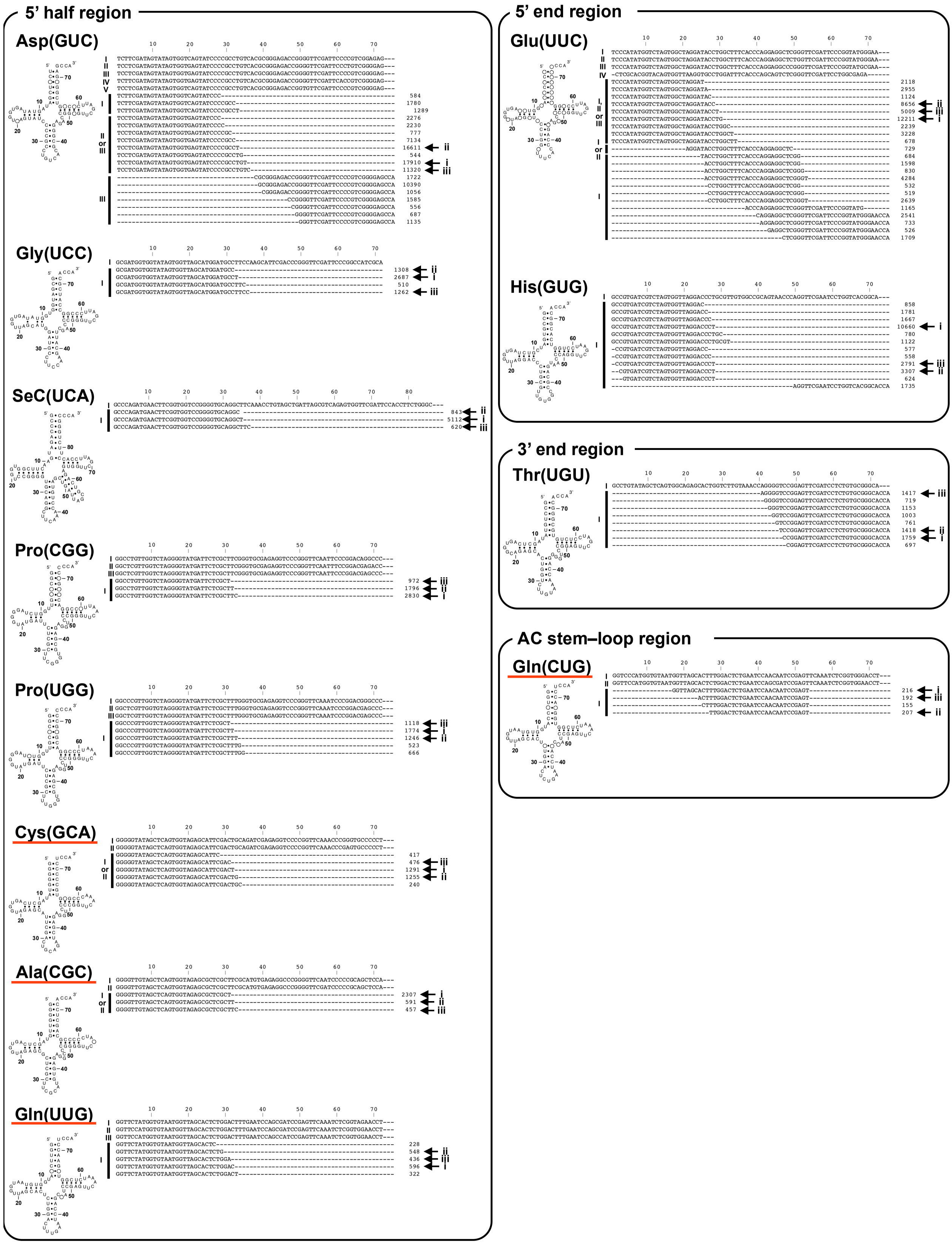

Figure S6. Another 12 examples of nuclear tRNAs and their tRFs in *T. cancriformis*. Titles in boxes represent the main tRF regions in each parental nuclear tRNA. Secondary structures of 12 mature nuclear tRNAs and nucleotide sequence alignments between these tRNAs and their tRFs are shown, as in Fig. 4. Highly expressed tRF reads were used for the sequence alignments (≥ 150 reads for Cys(GCA), Ala(CGC), Gln(UUG), and Gln(CUG), which are indicated with red lines, and ≥ 500 reads for the other anticodons). Top three read counts among the tRF variants are indicated with arrows and Roman numerals i–iii. tRNA gene subtypes (I–V) are also shown on the left of the sequence alignments.

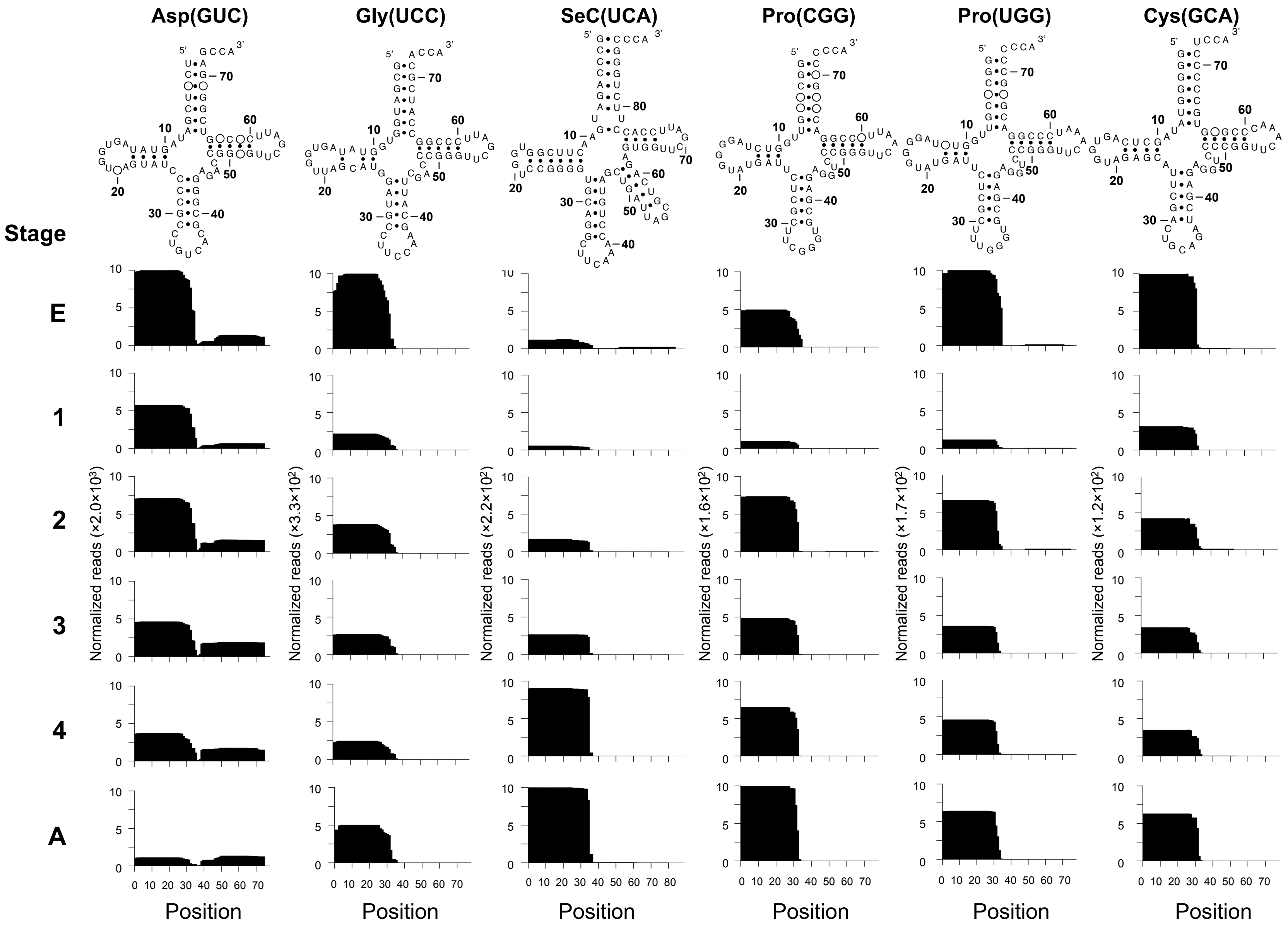

Figure S7. Expression of another 12 nuclear tRFs during *T. cancriformis* development (see Fig. 5). The accumulation of all tRF reads that mapped to the 12 individual parental nuclear tRNA sequences are shown for each of the six developmental stages.

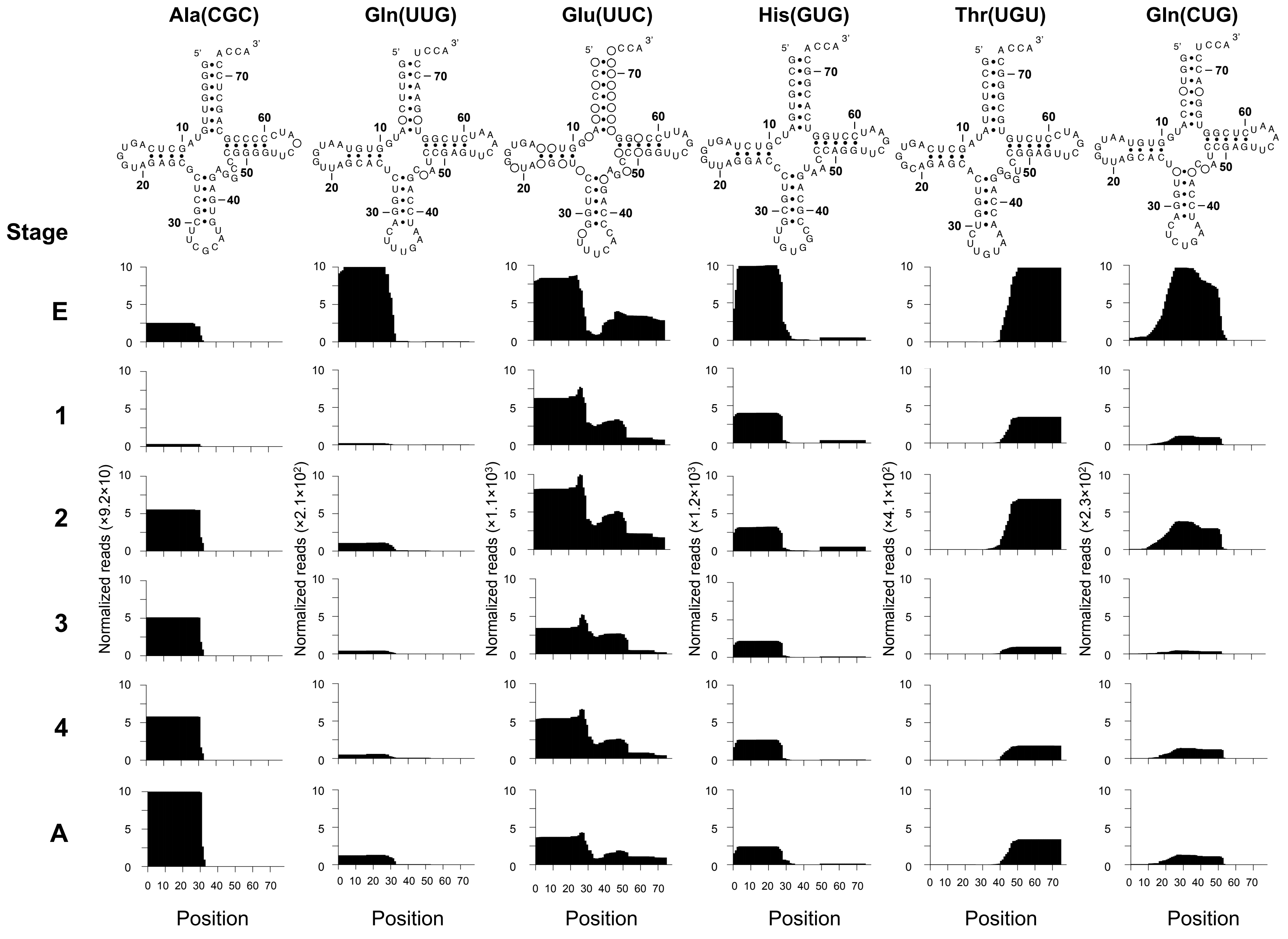

Figure S7 (Continued)
